# Occupational and non-occupational risk factors correlating with the severity of clinical manifestations of carpal tunnel syndrome and related work disability among workers who work with a computer

**DOI:** 10.2478/aiht-2023-74-3754

**Published:** 2023-12-29

**Authors:** Blerim Çupi, Ivana Šarac, Jovana J. Jovanović, Stefan Jovanović, Gordana Petrović-Oggiano, Jasmina Debeljak-Martačić, Jovica Jovanović

**Affiliations:** Besa Meditor Primary Healthcare Centre, Oslomej, Kičevo, North Macedonia; University of Niš Faculty of Medicine, Department of Occupational Health, Niš, Serbia; University of Belgrade Institute for Medical Research, National Institute of Republic of Serbia, Centre of Research Excellence in Nutrition and Metabolism, Belgrade, Serbia; University Clinical Centre Niš, Clinic of Plastic Surgery, Niš, Serbia; Institute of Occupational Medicine, Niš, Serbia

**Keywords:** Boston Carpal Tunnel Syndrome questionnaire, nerve compression, nerve conduction, occupational exposure, sick leave, bolovanje, Bostonski upitnik sindroma karpalnog tunela, profesionalna izloženost, provodljivost živca, sindromi kompresije živaca

## Abstract

The contribution of certain occupational and personal factors to the development of carpal tunnel syndrome (CTS) is still uncertain. We investigated which specific occupational and non-occupational factors correlate with the level of clinical manifestations and work disability related to CTS. The study included 190 workers who work with a computer and have diagnosed CTS (100 men, 90 women, aged 20–65 years). Subjective experience of CTS-related impairments was assessed with the Symptom Severity Scale (SSS) and the Functional Status Scale (FSS) of the Boston Carpal Tunnel Syndrome Questionnaire (BCTQ). The objective, neural impairments were tested with electrodiagnostics (EDX), whereas CTS-related work disability data were collected from medical records. We found a high inter-correlation between BCTQ, EDX, and work disability data. These also showed high correlations with certain occupational factors (duration of computer-working in months and hours spent daily in computer-working, certain ergonomic, microclimatic, and other occupational conditions) and non-occupational factors (demographic and lifestyle factors: nutritional status, diet, smoking, alcohol consumption, and physical activity). Despite its limitations, our study has identified occupational and non-occupational risk factors that can aggravate CTS and work disability, but which can also be improved with workplace and lifestyle preventive and corrective measures. More research is needed, though, to establish the possible causal relationships and the independent influence of each of those risk factors.

## Abstract (sl)

Doprinos pojedinih profesionalnih i osobnih čimbenika u nastanku sindroma karpalnog tunela (CTS) još uvijek je neizvjestan. Proučavali smo povezanost određenih profesionalnih i neprofesionalnih čimbenika s razinom kliničkih manifestacija i radne nesposobnosti u radnika s CTS-om koji rade na računalu. Tijekom redovitih pregleda službe medicine rada bilo je angažirano 190 radnika iz Sjeverne Makedonije s dijagnosticiranim CTS-om (100 muškaraca i 90 žena u dobi od 20 do 65 godina), koji rade na računalu. Razina subjektivnih simptoma povezanih s CTS-om procijenjena je ljestvicom ozbiljnosti simptoma (SSS) i ljestvicom funkcionalnog statusa (FSS) Bostonskoga upitnika sindroma karpalnog tunela (BCTQ); elektrodijagnostičko (EDX) testiranje korišteno je za procjenu razine objektivnih neuralnih oštećenja; podatci o radnoj nesposobnosti vezanoj za CTS prikupljeni su iz medicinske dokumentacije; razina profesionalnih i neprofesionalnih čimbenika procijenjena je putem posebno osmišljenog upitnika. Postojala je visoka međukorelacija između SSS, FSS, EDX rezultata i privremene/trajne nesposobnosti za rad vezane za CTS. Svi ti pokazatelji također su pokazali visoke korelacije s određenim profesionalnim čimbenicima (trajanje rada za računalom u mjesecima i vrijeme provedeno u radu za računalom tijekom dana, određeni ergonomski, mikroklimatski i drugi profesionalni uvjeti) i neprofesionalnim čimbenicima (demografski faktori i način života: prehranjenost, prehrana, pušenje, konzumacija alkohola, tjelesna aktivnost). Ova studija pokazuje da se brojni profesionalni i neprofesionalni čimbenici (od kojih se većina može mijenjati) mogu povezati s težinom kliničkih manifestacija i nesposobnošću za rad u vezi s CTS-om, što upućuje na potrebu pravodobne provedbe preventivnih i korektivnih mjera kako na radnome mjestu tako i u načinu života.

Many studies indicate that computer-operating workers run a higher risk of upper extremity musculoskeletal disorders, carpal tunnel syndrome (CTS) in particular (1, 2, 3, 4, 5, 6, 7, 8, 9, 10, 11). However, despite ample evidence, the association between work on computers and the development of CTS is still controversial, since the available data are inconclusive (19, 20, 21, 22, 23, 24, 25, 26, 27, 28). What seems to lack is an all-encompassing analysis of factors implicated in the development of CTS (3, 7, 8, 9, 10, 12, 13, 14, 15, 16, 17, 18). Apart from work-related long-term strain on the hands or wrists in awkward position, repetition, forceful hand/arm exertion, or vibration reported by a number of studies (29, 30, 31, 32, 33, 34), there are some non-occupational risks, such as genetics, female sex, age, obesity, diabetes, thyroid diseases, inflammatory arthritis, hand trauma, and pregnancy that can be involved in CTS development (35, 36, 37, 38, 39).

The aim of our study was therefore to take a comprehensive look at occupational and non-occupational risk factors associated with CTS and how they affect computer operators. Since information on work disability related to CTS among computer-operating workers are scarce, we also wanted to take a better look at factors that contribute to temporary and permanent disability (2).

## MATERIALS AND METHODS

### Study participants

The study included 190 workers aged 20–65 years (100 men, 90 women) who were previously diagnosed with CTS and who worked with computers at least 1 h per day. In February 2023, these participants took their regular occupational medicine check-up at the Primary Health Care Institution Besa Meditor, Oslomej, Kičevo, North Macedonia. They came from different business sectors (from local administration to industry clerks in the thermal power and solar power plants, and automobile industry). The check-up did not include CTS diagnosis, as it had been established earlier by clinical specialists according to clinical guidelines (40, 41).

All participants signed an informed consent to participate, and the study was conducted in accordance with the Declaration of Helsinki and approved by the Ethics Committee of the University of Niš Faculty of Medicine, Niš, Serbia (Decision No. 12-1285/2-1 of 2 February 2023).

To make our research as comprehensive as possible, we included a wide variety of participants information collected through a specifically designed questionnaire and medical documentation: sociodemographic data, medical history of CTS and other diseases, Boston Carpal Tunnel Questionnaire (BCTQ) score, electrodiagnostic (EDX) test results, work disability data, occupational factors, diet, physical activity, anthropometric data, and laboratory results.

### Boston Carpal Tunnel Questionnaire (BCTQ)

All participants completed the BCTQ (42) version translated into Macedonian, which consists of the Symptom Severity Scale (SSS) and the Functional Status Scale (FSS). The first consists of 11 items through which participants rate the severity of pain, paraesthesia, numbness, weakness, nocturnal symptoms, and difficulty grasping objects. The second consists of eight items through which participants rate hand function difficulties in writing, buttoning, holding a book while reading, gripping a telephone handle, opening jars, performing household chores, carrying grocery bags, bathing/taking shower, and dressing (42, 43). Each item is scored one (no symptoms/difficulties) to five (worst symptoms/cannot perform the activity at all) and the total given as mean score for each scale. Higher scores indicate severe symptoms (SSS) or impaired function (FSS) (43).

### Electrodiagnostic (EDX) testing

Electrophysiological testing was run on the Medelec-Synergy EMG instrument (Oxford Instruments, Surrey, UK) by a single specialist of physical medicine. Standardised measurements included motor and fibre conduction velocities and F-wave latencies of the median and ulnar nerves as described elsewhere (44).

Briefly, motor nerve conduction velocity was measured using the orthodromic technique by placing percutaneous detection electrodes on the *abductor pollicis brevis* (for *n. medianus*) and on the *abductor digiti minimi* (for *n. ulnaris*) and stimulating the motor points of the nerves with supramaximal intensity on the wrist, 8 cm proximal to the detection electrodes.

Sensory nerve conduction velocity was measured using the antidromic technique by stimulating the wrist nerves with electrodes and taking readings with ring electrodes placed on the index finger (for *n. medianus*) or little finger (*for n. ulnaris*), 14 cm distal to the stimulating electrode.

To control for confounding factors of age, temperature, illness (i.e., diabetes), gender, and hand size, sensory distal onset latency was measured on the ring finger by stimulating both the median and ulnar nerve at the level of the wrist, 14 cm proximal to the detection ring electrode. In case both hands were affected by CTS, we show the measurements on the more affected hand.

### Work disability data

Data evidencing temporary work disability related to CTS and its duration (in days) over the last year were obtained from participants’ GP medical records, while data on recommendations for a job change and applications for permanent work disability related to CTS were obtained from participants and related medical documentation during their occupational-medicine check-ups.

### Assessment of occupational factors

Our assessment was divided between two large groups of factors: those associated and those not associated with working on a computer. Details are available in the Results section. Most data were self-reported and collected through a specifically designed questionnaire but microclimatic factors and vibrations at the workplace were measured by specialists of occupational medicine and safety at work as described elsewhere (45, 46). General job satisfaction was self-rated by the participants as “poorly satisfied”, “moderately satisfied”, or “very satisfied”.

### Anthropometric and blood pressure measurements

Participants’ body height and weight were measured by trained medical nurses at 8 am on empty stomach and bladder, wearing only underwear. Body mass index (BMI) was calculated, and the participants classified in three groups: normal weight (BMI: 18.5–24.9 kg/m^2^), overweight (BMI: 25–29.9 kg/m^2^), and obese (BMI: >30 kg/m^2^) (47).

Arterial blood pressure was measured on the left upper arm after sitting for at least 10 min with a calibrated mechanic sphygmomanometer and stethoscope (Becton Dickinson, Franklin Lakes, NJ, United States). Hypertension was determined in participants with systolic pressure ≥140 mm Hg and/or diastolic blood pressure ≥90 mm Hg (48).

### Assessment of dietary habits and physical activity

Data on the frequency of consumption of certain food groups and dietary supplements over the past year and level of physical activity over the past week were collected by a trained medical doctor using a food propensity questionnaire (FPQ) in accordance with the European Food Safety Authority (EFSA) EU Menu methodology (49, 50) and the International Physical Activity Questionnaire – Short Form (IPAQ-SF) (51). Data on the frequency of consumption of certain food groups and dietary supplements over the past year were categorised into seven categories: 1) never; 2) less than once a month; 3) one to three times a month; 4) once a week; 5) two to three times a week; 6) four to five times a week; and 7) six to seven times a week. Metabolic equivalents (METs) (expressed in minutes per week) were calculated as described in detail by Craig et al. (51).

### Blood sampling and laboratory biochemistry

Fasting blood samples were collected between 7 and 8 am. Erythrocyte sedimentation rates (ESR) were analysed with the Westergren's method at 18 °C and the results show one-hour values. Fibrinogen was measured with the Clauss fibrinogen assay (52). Standard biochemical assays were run on an ARCHITECT c8000 Abbott clinical chemistry analyser (Abbott Laboratories, Abbott Park, IL, USA) using commercially available kits (Abbott Laboratories) and included serum glucose, total cholesterol, high-density lipoproteins (HDL)-cholesterol and low-density lipoproteins (LDL)-cholesterol, triglycerides, urea, creatinine, aspartate aminotransferase (AST), alanine aminotransferase (ALT), gamma-glutamyl transferase (GGT), uric acid, and C-reactive protein (CRP). Intra- and inter-assay coefficients of variation (CVs) for all measurements were <6 %.

### Statistical analysis

The required minimum sample size for Spearman correlation analyses with meaningful correlation coefficients and moderate effect (*r_s_*≥0.300) was 85 participants, and for the correlation analyses with correlation coefficients *r_s_*≥0.600 it was 20 participants, when the level of significance was set to 5 % (α=0.05) and the study power was set to 80 % (β=0.20) (53). However, since we had multiple testing, we had to increase the number of the subjects included (54).

The normality of continuous data distribution was tested with the Kolmogorov-Smirnov test. Since no continuous data had normal distribution, nominal/categorical data are presented either as a number and percent, while all other data as median and interquartile range.

Correlations between different occupational and non-occupational factors with the severity of subjective symptoms (SSS and FSS scales), objective impairments (EDX results), and temporary and permanent work disability indicators were assessed using the Spearman's *r_s_* correlation coefficient (to assess correlations between two non-parametrically distributed numeric or ordinal variables), Glass rank-biserial *r_rb_* correlation coefficient (to assess correlations between one non-parametrically distributed numeric or ordinal variable and one dichotomous categorical variable) (55, 56, 57), and the phi *φ* or Cramér's V *φ_c_* correlation coefficients (to assess correlations between two categorical variables).

Two categories (groups) were compared using the Mann-Whitney *U* and chi-squared test.

For all analyses, statistical significance was assumed at a two-tailed p<0.05, but also the Bonferroni correction for multiple testing was applied (58). All analyses were run on the SPSS 22.0 software (SPSS Inc., Chicago, IL, United States).

## RESULTS

### Descriptive statistics

Detailed demographic, anthropometric, overall health, and lifestyle characteristics of the study participants are given in Table 1. Interestingly, most are overweight or obese, have quite an unhealthy lifestyle (including smoking, high alcohol consumption, physical inactivity), and suffer from hypertension and endocrine glands disorders (diabetes or thyroid disease).

**Table 1:** Demographic, anthropometric, health, and lifestyle characteristics of the study participants (N=190)

**Variable**	**All subjects (N=190)**
Male gender, n (%)	90 (47.4)
Female gender, n (%)	100 (52.6)
Age, years, median (IQR)	36.0 (28.8–51.0)
<35 years old, n (%)	79 (41.6)
35–50 years old, n (%)	63 (33.2)
>50 years old, n (%)	48 (25.3)
Undergraduate education (high school) (n %)	106 (55.8)
Graduate education (university) (n %)	79 (41.6)
Postgraduate education (n %)	5 (2.6)
Right hand dominant, n (%)	176 (92.6)
Left hand dominant, n (%)	14 (7.4)
Body mass index, kg/m^2^, median (IQR)	26.0 (23.8–27.8)
Normal-weight, n (%)	75 (39.5)
Overweight, n (%)	96 (50.5)
Obese, n (%)	19 (10.0)
Systolic blood pressure, mm Hg, median (IQR)	145.0 (125.0–155.0)
Diastolic blood pressure, mm Hg, median (IQR)	95.0 (80.0–105.0)
Hypertension, n (%)	111 (58.4)
Other cardiovascular diseases (apart from hypertension), n (%)	5 (2.6)
Diseases of endocrine glands (diabetes, thyroid disease), n (%)	96 (50.5)
Diseases of the blood and blood-forming organs, n (%)	8 (4.2)
Mental disorders, n (%)	1 (0.5)
Diseases of the genitourinary system, n (%)	4 (2.1)
Other diseases of the musculoskeletal system unrelated to CTS, n (%)	20 (10.5)
Never smoker / former smoker, n (%)	49 (25.8)
Current smoker, n (%)	141 (74.2)
<10 cigarettes a day, n (%)	20 (10.5)
10–20 cigarettes a day, n (%)	52 (27.4)
>20 cigarettes a day, n (%)	69 (36.3)
No alcohol consumption, n (%)	63 (33.2)
Moderate alcohol consumption, n (%)	68 (35.8)
Occasional alcohol overconsumption, n (%)	51 (26.8)
Frequent alcohol overconsumption, n (%)	8 (4.2)
Low physical activity, n (%)	138 (72.6)
Intermediate physical activity, n (%)	41 (21.6)
High physical activity, n (%)	11 (5.8)
Physical activity METs scores, min week^−1^, median (IQR)	0.0 (0.0–1512.0)

IQR – interquartile range; METs – metabolic equivalents

Table 2 shows participants’ medical history related to CTS, including subjective (BCSQ) and objective (EDX) impairments and work disability.

**Table 2:** Medical history related to CTS, subjective and objective impairments (BCSQ and EDX results) and work disability indicators

**Variable**	**All (N=190)**
Duration of CTS since diagnosis, months, median (IQR)	40.0 (15.0–130.0)
**Affected hand, n (%)**	
Dominant	88 (46.3)
Non-dominant	18 (9.5)
Bilateral	84 (44.2)
Family history of CTS, yes, n (%)	96 (50.5)
**Treatment, n (%)**	
Medication only	18 (9.5)
Physical therapy included, without surgery	106 (55.8)
Surgery included	66 (34.7)
Steroidal anti-inflammatory medication included, n (%)	103 (54.2)
Non-steroidal anti-inflammatory medication included, n (%)	125 (65.8)
**Boston Carpal Tunnel Questionnaire (BCTQ)**	
Symptom Severity Scale (SSS) score, range 1–5, median (IQR)	2.9 (1.0–4.1)
Functional Status Scale (FSS) score, range 1–5, median (IQR)	2.6 (1.0–4.1)
**Electrodiagnostic (EDX) testing**	
Motor conduction velocity (MCV) of n. *medianus*, m/s, median (IQR)	49.5 (41.0–57.0)
Sensory conduction velocity (SCV) of n. *medianus*, m/s, median (IQR)	50.0 (41.0–58.0)
Sensory distal onset latency (SDOL) of n. *medianus*, ms, median (IQR)	1.2 (0.8–2.0)
Motor conduction velocity (MCV) of n. *ulnaris*, m/s, median (IQR)	58.0 (57.0–60.0)
Sensory conduction velocity (SCV) of n. *ulnaris*, m/s, median (IQR)	59.0 (58.0–61.0)
Sensory distal onset latency (SDOL) of n. *unaris*, ms, median (IQR)	1.6 (1.5–1.6)
Ratio of distal sensory latency (SDOL-R) of n. *medianus* and n. *unaris*, median (IQR)	0.8 (0.5–1.3)
**Work disability indicators**	
Duration of temporary work disability related to CTS during the preceding year, days, median (IQR)	31.5 (14.0–50.0)
Applications for permanent work disability because of CTS, n (%)	62 (32.6)
Job changes because of CTS, n (%)	75 (39.5)

BCTQ – Boston Carpal Tunnel Questionnaire; CTS – carpal tunnel syndrome; EDX – electrodiagnostic testing; FSS – Functional Status Scale; IQR – interquartile range; MCV – motor conduction velocity; SCV – sensory conduction velocity; SDOL – sensory distal onset latency; SDOL-R – ratio of sensory distal onset latency

All the participants met the criteria for the EDX diagnosis of CTS in active workers or general population (44), while data from BCSQ indicated a moderate handicap (59, 60). All the participants also reported temporary work disability related to CTS during the preceding year, with the median duration of about one month (ranging from 2 to 85 days), and 62 applied for permanent (long-term) work disability pension because of CTS (on personal request, request of the employer, or request of medical commission due to prolonged work disability), but only 10 were approved preterm pension after this study was completed. Still, about 40 % received a job change recommendation because of CTS after medical checkup by a specialist of occupational medicine. The 62 participants who had applied for permanent work disability were older (median age: 55 years), with longer duration of disease (median: 13.1 years), worked longer with computer during their career (median: 15.4 years), spent more hours on work with computer (median: 6.2 h a day), and were on longer sick leaves in the past year (median; 55.5 days) compared to those who had never applied for disability pension (Mann-Whitney *U* test, p<0.001 for all).

Table 3 shows specific data about occupational factors, both related and unrelated to computer work. Taken as a whole, the participants spent 4.4 years (ranging from 1 month to 20 years) working on a computer for 4 h a day at work (ranging from 1 h to 6.8 h) and 1 h a day in their free time (ranging from 5 min to 3 h). Although the overall median time spent on a computer is moderate, the wide variety of ranges allowed us to analyse correlations between the duration of computer work and severity of CTS impairments.

**Table 3:** Participants’ data on occupational factors related and not related to computer-work

**Variable**	**All (N=190)**
Duration of occupational work with computer, months, median (IQR)	52.5 (27.8–150.0)
Average daily time spent in occupational work with computer, minutes, median (IQR)	240.0 (170.0–360.0)
Average daily time spent in non-occupational work with computer, minutes, median (IQR)	60.0 (40.0–120.0)
Number of additional breaks while occupationally working with computer (excluding the legal one of 30 minutes), median (IQR)	2.0 (2.0–4.0)
Total duration of additional breaks while occupationally working with computer, minutes, median (IQR)	6.0 (6.0–12.0)
Exercises (finger and thumb stretching and wrist and hand joints rotations) during breaks at work, n (%)	80 (42.1)
Exercises (finger and thumb stretching and wrist and hand joints rotations) during breaks outside of work, n (%)	71 (37.4)
Use of a mouse while occupationally working with computer, n (%)	190 (100.0)
Use of a mouse pad while occupationally working with computer, n (%)	162 (85.3)
Use of an ergonomically designed mouse, mouse pad, and keyboard (adapted to the anatomical characteristics of the user's hand) while occupationally working with computer, n (%)	74 (38.9)
Completed training for safe and healthy work with computers, n (%)	77 (40.5)
Other repetitive jobs with hands at the workplace not related to computer-work, n (%)	99 (52.1)
Other repetitive jobs with hands outside the workplace, n (%)	44 (23.2)
Heavy physical exertion with arms/hands at the workplace outside the computer-work, n (%)	28 (14.7)
Heavy physical exertion with arms/hands outside the workplace, n (%)	33 (17.4)
Exposure to vibrations at the workplace, n (%)	96 (50.5)
Duration of exposure to vibrations at the workplace, minutes, median (IQR)	10.0 (0.0–30.0)
The level of vibration at the workplace above >5 m/s^2^ A(8), n (%)	5 (2.6)
Exposure to vibrations outside the workplace, n (%)	57 (30.0)
**Air temperature at the workplace, n (%)**	
Normal	156 (82.1)
High	3 (1.6)
Low	31 (16.3)
Air temperature at the workplace, °C, median (IQR)	23.0 (18.0–26.0)
**Relative air humidity at the workplace, n (%)**	
Normal	91 (47.9)
Low	3 (1.6)
High	96 (50.5)
**Air-flow speed at the workplace, n (%)**	
Normal	92 (48.4)
Low	3 (1.6)
High	95 (50.0)
**General job satisfaction, n (%)**	
Poorly satisfied	111 (58.4)
Moderately satisfied	66 (34.7)
Very satisfied	13 (6.8)

IQR – interquartile range

An interesting finding is that the total duration of work with computer (in months, hours, or both) negatively correlates with the use of ergonomically designed devices, hand exercises, and training for safe and healthy work with computers reported by 40 % of the participants (p<0.001 for all, data not shown), indicating that new workers and those spending less time on computers had better training and practiced ergonomic measures better.

Heavy physical exertion with arms/hands at work was not performed by many participants, but other repetitive jobs with hands in addition to computer-work were reported by about half of them. About half the participants also reported occupational exposure to vibrations lasting between 10 min and 40 min, but only five participants were exposed to vibrations above the regulatory exposure limit of 5 m/s^2^ in average for eight-hour work, while about 30.0 % were also exposed to vibrations outside of work. Curiously enough, the total duration of work with computer highly correlates with the duration of exposure to vibrations, heavy physical exertion, other repetitive movements, and negative microclimatic conditions (p<0.001 for all, data not shown).

For the sake of easy viewing, Table 4 shows food and dietary supplement consumption in three categories of frequency, although originally there were seven (see the Methods section). The vast majority of the participants reported quite unhealthy eating habits, such as frequent consumption of refined grain products, red and processed meat, tallow, sweets and dried fruits, alcoholic beverages (spirits in particular), and carbonated sugar-added beverages. In contrast, the reported consumption of dairy products, eggs, poultry meat, fish and sea food, integral grains, fresh vegetables and fruits, nuts, vegetable oils (including sunflower oil and olive oil), and pork lard was surprisingly low. Between 55 % and 65 % of the participants reported not to consume any dietary supplements, and those who consumed them show no particular preference.

**Table 4:** Dietary habits and consumption of food supplements among study participants

**Food groups / Dietary supplements**		**Frequency**		
		**Never or less than once a month**	**Once a month to once a week**	**Two of more times a week**
		**All (N=190)**		
**Food groups**				
Consumes refined grain products (e.g., white bread, pasta, puff pastry) in his/her diet, n (%)		23 (12.1)	43 (22.6)	124 (65.3)
Consumes integral grain products (e.g., integral bread, flakes, muesli) in his/her diet, n (%)		137 (72.1)	19 (10.0)	34 (17.9)
Consumes dairy products in his diet, n (%)		23 (12.1)	98 (51.6)	69 (36.3)
Consumes eggs in his/her diet, n (%)		123 (64.7)	31 (16.30)	36 (18.9)
Consumes poultry meat in his/her diet, n (%)		123 (64.7)	35 (18.4)	32 (16.8)
Consumes red and processed meat in his/her diet, n (%)		27 (14.2)	72 (37.9)	91 (47.9)
Consumes fish and seafood in his/her diet, n (%)		132 (69.5)	31 (16.3)	27 (14.2)
Consumes fresh vegetables (in salad) in his/her diet, n (%)		140 (73.7)	17 (8.9)	33 (17.4)
Consumes legumes (e.g., beans, peas, lentils, green beans) in his/her diet, n (%)		41 (21.6)	75 (39.5)	74 (38.9)
Consumes fresh fruit in his/her diet, n (%)		136 (71.6)	20 (10.5)	34 (17.9)
Consumes nuts (e.g., almonds, walnuts, hazelnuts) in his/her diet, n (%)		129 (67.9)	30 (15.8)	31 (16.3)
Consumes sunflower oil in his/her diet, n (%)		127 (66.8)	37 (19.5)	26 (13.7)
Consumes olive oil in his/her diet, n (%)		144 (75.8)	13 (6.8)	33 (17.4)
Consumes pork lard in his/her diet, n (%)		148 (77.9)	24 (12.6)	18 (9.5)
Consumes tallow in his/her diet, n (%)		4 (2.1)	19 (10.0)	167 (87.9)
Consumes sweets in his/her diet, n (%)		27 (14.2)	25 (13.2)	138 (72.6)
Consumes dry fruits in his/her diet, n (%)		25 (13.2)	53 (27.9)	112 (58.9)
Consumes spirits in his/her diet, n (%)		32 (16.8)	36 (18.9)	122 (64.2)
Consumes beer in his/her diet, n (%)		136 (71.6)	19 (10.0)	35 (18.4)
Consumes wine in his/her diet, n (%)		53 (27.9)	86 (45.3)	51 (26.8)
Consumes carbonated drinks with sugar and energy drinks in his/her diet, n (%)		40 (21.1)	29 (15.3)	121 (63.7)
Consumes freshly squeezed juices in his/her diet, n (%)		138 (72.6)	18 (9.5)	34 (17.9)
**Dietary supplements**				
Consumes vitamin A supplements, n (%)		91 (47.9)	62 (32.6)	37 (19.5)
Consumes vitamin D supplements, n (%)		109 (57.4)	50 (26.3)	31 (16.3)
Consumes vitamin E supplements, n (%)		113 (59.5)	47 (24.7)	30 (15.8)
Consumes vitamin C supplements, n (%)		106 (55.8)	55 (28.9)	29 (15.3)
Consumes vitamin B group supplements, n (%)		112 (58.9)	44 (23.2)	34 (17.9)
Consumes folic acid supplements, n (%)		120 (63.2)	46 (24.2)	24 (12.6)
Consumes calcium supplements, n (%)		113 (59.5)	48 (25.3)	29 (15.3)
Consumes magnesium supplements, n (%)		114 (60.0)	52 (27.4)	24 (12.6)
Consumes iron supplements, n (%)		118 (62.1)	63 (33.2)	9 (4.7)
Consumes zinc supplements, n (%)		119 (62.6)	45 (23.7)	26 (13.7)
Consumes selenium supplements, n (%)		123 (64.7)	41 (21.6)	26 (13.7)
Consumes fish oil omega-3 supplements, n (%)		113 (59.5)	42 (22.1)	35 (18.4)
Consumes probiotic preparations, n (%)		113 (59.5)	39 (20.5)	38 (20.0)

IQR – interquartile range

Our correlation analysis shows that all dietary factors (except for dairy products, legumes, and wine) and consumption of supplements highly inter-correlate and tend to cluster in two opposite eating patterns: a) the predominant “unhealthy” one with high consumption of refined grains, red and processed meat, tallow, sweets, dried fruits, spirits, and carbonated beverages (“dietary cluster 1”) and very low consumption of integral grains, eggs, poultry, fish/sea products, fresh vegetables and fruits, fruit-juices, nuts, vegetable oils, pork lard, and beer (“dietary cluster 2”) and supplements and b) the reverse “more healthy” pattern, which was much rarer. The food groups from one cluster highly positively correlate within the same cluster and highly negatively correlate with the food groups from the other cluster (p<0.001 for all, data not shown).

The laboratory data seem to reflect the anthropometric measurements and reports of dietary and physical (in)activity habits, as most participants showed values indicative of metabolic syndrome and inflammation: low HDL- and high LDL-cholesterol, triglycerides, glucose, ALT, AST, GGT, uric acid, urea, creatinine, fibrinogen, CRP, and sedimentation (Table 5). In fact, we found significant correlations between all laboratory indicators of metabolic risk and inflammation (except AST) and anthropometric and dietary data, smoking, alcohol use, physical inactivity, and hypertension (p<0.001 for all, data not shown).

**Table 5:** Biochemical laboratory markers of inflammation and metabolic risk among study participants

**Variable**	**All (N=190)**
LDL-cholesterol, mmol/L, median (IQR)	3.6 (2.7–4.4)
HDL-cholesterol, mmol/L, median (IQR)	0.9 (0.7–1.3)
Triglycerides, mmol/L, median (IQR)	1.7 (1.4–1.9)
Glucose, mmol/L, median (IQR)	5.6 (4.9–6.3)
AST, IU/L, median (IQR)	35.5 (20.0–41.0)
ALT, IU/L, median (IQR)	41.0 (35.0–51.3)
GGT, IU/L, median (IQR)	47.0 (38.0–58.0)
Uric acid, mmol/L, median (IQR)	416.0 (285.0–430.0)
Urea, mmol/L, median (IQR)	8.5 (6.5–9.3)
Creatinine, mmol/L, median (IQR)	92.0 (49.5–102.4)
ESR, mm/h, median (IQR)	20.0 (15.0–28.0)
Fibrinogen, g/L, median (IQR)	4.0 (3.2–4.7)
CRP, mg/L, median (IQR)	5.7 (4.0–7.9)

ALT – alanine aminotransferase; AST – aspartate aminotransferase; CRP – C-reactive protein; ESR – erythrocyte sedimentation rate; GGT – gamma-glutamyl transferase; HDL – high-density lipoprotein; IQR – interquartile range; LDL – low-density lipoprotein

### Correlations with CTS

Tables 6–10 show the findings of our correlation analysis between occupational and non-occupational factors and the severity of CTS-related clinical manifestations and work disability. Since many variables were included in the study, we applied the Bonferroni correction. Significant correlations (p<0.00003472) obtained this way are underlined in the tables.

**Table 6:** Inter-correlations of indicators of subjective and objective impairments (BCSQ and EDX results) and work disability related to CTS, and their correlation with other clinical characteristics of CTS and personal (demographic, anthropometric, overall health status and lifestyle) factors in the cohort of workers who work with a computer (N=190)

**Variable**	**Boston Carpal Tunnel Questionnaire (BCTQ)**		**Electrodiagnostic (EDX) testing**							**Work disability related to CTS**		
	**SSS score**	**FSS score**	**Median nerve MCV**	**Median nerve SCV**	**Median nerve SDOL**	**Ulnar nerve MCV**	**Ulnar nerve SCV**	**Ulnar nerve SDOL**	**Median to ulnar nerve SDOL-R**	**Temporary work disability duration**	**Permanent work disability application**	**Recommended job change**
	*r_s_ / r_rb_* ^§^	*r_s_ / r_rb_* ^§^	*r_s_ / r_rb_* ^§^	*r_s_ / r_rb_* ^§^	*r_s_ / r_rb_* ^§^	*r_s_ / r_rb_* ^§^	*r_s_ / r_rb_* ^§^	*r_s_ / r_rb_* ^§^	*r_s_ / r_rb_* ^§^	*r_s_ / r_rb_* ^§^	*r_rb_ / φ ^#^*	*r_rb_ / φ ^#^*
**Boston Carpal Tunnel Questionnaire (BCTQ)**												
Symptom Severity Scale (SSS) score	-	0.944^**^	−0.840^**^	−0.840^**^	0.842^**^	−0.230^**^	−0.218^**^	0.416^**^	0.777^**^	0.841^**^	0.697^**^	0.708^**^
Functional Status Scale (FSS) score	0.944^**^	-	−0.856^**^	−0.853^**^	0.851^**^	−0.205^**^	−0.194^**^	0.422^**^	0.792^**^	0.856^**^	0.688^**^	0.722^**^
**Electrodiagnostic (EDX) testing**												
Median motor conduction velocity (MCV) m/s	−0.840^**^	−0.856^**^	-	0.998^**^	−0.984^**^	0.207^**^	0.187^**^	−0.428^**^	−0.899^**^	−0.986^**^	−0.695^**^	−0.676^**^
Median sensory conduction velocity (SCV) m/s	−0.840^**^	−0.853^**^	0.998^**^	-	−0.984^**^	0.204^**^	0.183^**^	−0.428^**^	−0.901^**^	−0.985^**^	−0.694^**^	−0.677^**^
Median sensory distal onset latency (SDOL) on ring finger, ms	0.842^**^	0.851^**^	−0.984^**^	−0.984^**^	-	−0.210^**^	−0.194^**^	0.422^**^	0.907^**^	0.985^**^	0.699^**^	0.669^**^
Ulnar motor conduction velocity (MCV), m/s	−0.230^**^	−0.205^**^	0.207^**^	0.204^**^	−0.210^**^	-	0.984^**^	−0.161^*^	−0.234^**^	−0.200^**^	−0,072	−0.170^**^
Ulnar sensory conduction velocity (SCV) m/s	−0.218^**^	−0.194^**^	0.187^**^	0.183^**^	−0.194^**^	0.984^**^	-	−0.164^*^	−0.224^**^	−0.184^**^	−0,063	−0.156^*^
Ulnar sensory distal onset latency (SDOL) on ring finger, ms	0.416^**^	0.422^**^	−0.428^**^	−0.428^**^	0.422^**^	−0.161^*^	−0.164^*^	-	0.378^**^	0.397^**^	0.270^**^	0.459^**^
Median to ulnar ratio of sensory distal onset latency (SDOL-R) on ring finger	0.777^**^	0.792^**^	−0.899^**^	−0.901^**^	0.907^**^	−0.234^**^	−0.224^**^	0.378^**^	-	0.894^**^	0.634^**^	0.703^**^
**Work disability indicators**												
Duration of temporary work disability related to CTS, days	0.841^**^	0.856^**^	−0.986^**^	−0.985^**^	0.985^**^	−0.200^**^	−0.184^**^	0.397^**^	0.894^**^	-	0.702^**^	0.673^**^
Applications for permanent work disability because of CTS	0.697^**^	0.688^**^	−0.695^**^	−0.694^**^	0.699^**^	−0.072	−0.063	0.270^**^	0.634^**^	0.702^**^	-	0.678^**^
Recommended job change because of CTS	0.708^**^	0.722^**^	−0.676^**^	−0.677^**^	0.669^**^	−0.170^**^	−0.156^*^	0.459^**^	0.703^**^	0.673^**^	0.678^**^	-
**Other clinical characteristics**												
Duration of CTS since diagnosis, months	0.843^**^	0.857^**^	−0.985^**^	−0.984^**^	0.979^**^	−0.206^**^	−0.188^**^	0.424^**^	0.888^**^	0.989^**^	0.684^**^	0.680^**^
Bilateral presentation	0.419^**^	0.420^**^	−0.334^**^	−0.330^**^	0.334^**^	−0.211^**^	−0.197^**^	0.187^**^	0.300^**^	0.332^**^	0.307^**^	0.343^**^
Family history for CTS	0.438^**^	0.446^**^	−0.379^**^	−0.372^**^	0.395^**^	−0.284^**^	−0.275^**^	0.293^**^	0.409^**^	0.387^**^	0.397^**^	0.498^**^
Treatment options (medication, physical therapy included, or also surgery included)	0.512^**^	0.500^**^	−0.416^**^	−0.417^**^	0.426^**^	−0.168^*^	−0.162^*^	0.300^**^	0.427^**^	0.424^**^	0.296^**^	0.399^**^
Steroidal anti-inflammatory medication included	0.673^**^	0.668^**^	−0.585^**^	−0.581^**^	0.582^**^	−0.264^**^	−0.253^**^	0.399^**^	0.570^**^	0.586^**^	0.550^**^	0.742^**^
Non-steroidal anti-inflammatory medication included	0.587^**^	0.578^**^	−0.590^**^	−0.584^**^	0.581^**^	−0.244^**^	−0.231^**^	0.320^**^	0.500^**^	0.584^**^	0.478^**^	0.582^**^
Medication only	−0.290^**^	−0.286^**^	0.156^*^	0.159^*^	−0.176^*^	0.015	0.024	−0.041	−0.203^**^	−0.175^*^	−0.225^**^	−0.261^**^
Physical therapy included	−0.076	−0.065	0.062	0.063	−0.056	0.093	0.082	−0.171^*^	−0.081	−0.049	0.054	0.003
Surgery included	0.257^**^	0.243^**^	−0.161^*^	−0.164^*^	0.167^*^	−0.107	−0.101	0.203^**^	0.209^**^	0.159^*^	0.082	0.157^*^
**Personal demographic, anthropometric, overall health status and lifestyle factors**												
Female gender	0.633^**^	0.637^**^	−0.494^**^	−0.493^**^	0.480^**^	−0.246^**^	−0.239^**^	0.343^**^	0.541^**^	0.482^**^	0.480^**^	0.723^**^
Age, years	0.836^**^	0.850^**^	−0.985^**^	−0.984^**^	0.987^**^	−0.212^**^	−0.194^**^	0.412^**^	0.909^**^	0.990^**^	0.681^**^	0.670^**^
Educational status	−0.295^**^	−0.270^**^	0.259^**^	0.266^**^	−0.265^**^	−0.003	−0.012	−0.018	−0.276^**^	−0.243^**^	−0.299^**^	−0.294^**^
Undergraduate (high school)	0.299^**^	0.277^**^	−0.272^**^	−0.279^**^	0.276^**^	0.001	0.007	0.022	0.290^**^	0.254^**^	0.303^**^	0.285^**^
Graduate (university)	−0.286^**^	−0.270^**^	0.281^**^	0.287^**^	−0.280^**^	0.005	0.003	−0.028	−0.301^**^	−0.259^**^	−0.291^**^	−0.244^**^
Postgraduate	−0.048	−0.026	−0.023	−0.017	0.008	−0.019	−0.032	0.018	0.027	0.012	−0.044	−0.133^*^
Body mass index, kg/m^2^	0.803^**^	0.805^**^	−0.835^**^	−0.834^**^	0.831^**^	−0.145^*^	−0.138^*^	0.303^**^	0.765^**^	0.838^**^	0.640^**^	0.611^**^
Systolic blood pressure, mm Hg	0.736^**^	0.767^**^	−0.687^**^	−0.685^**^	0.697^**^	−0.271^**^	−0.256^**^	0.380^**^	0.659^**^	0.700^**^	0.585^**^	0.715^**^
Diastolic blood pressure, mm Hg	0.736^**^	0.757^**^	−0.690^**^	−0.689^**^	0.697^**^	−0.269^**^	−0.259^**^	0.365^**^	0.679^**^	0.706^**^	0.596^**^	0.719^**^
Other diseases	0.593^**^	0.613^**^	−0.435^**^	−0.429^**^	0.447^**^	−0.149^*^	−0.139^*^	0.166^*^	0.446^**^	0.455^**^	0.425^**^	0.522^**^
Diseases of endocrine glands (diabetes, thyroid disease)	0.515^**^	0.524^**^	−0.365^**^	−0.359^**^	0.365^**^	−0.255^**^	−0.262^**^	0.172^**^	0.348^**^	0.375^**^	0.419^**^	0.433^**^
Smoking habit	0.630^**^	0.631^**^	−0.459^**^	−0.456^**^	0.458^**^	−0.077	−0.075	0.250^**^	0.420^**^	0.472^**^	0.492^**^	0.597^**^
Alcohol consumption	0.686^**^	0.730^**^	−0.621^**^	−0.612^**^	0.626^**^	−0.149^*^	−0.151^*^	0.320^**^	0.591^**^	0.636^**^	0.547^**^	0.665^**^
Physical activity level	−0.605^**^	−0.621^**^	0.753^**^	0.752^**^	−0.750^**^	0.306^**^	0.320^**^	−0.392^**^	−0.707^**^	−0.753^**^	−0.424^**^	−0.492^**^
Physical activity METs scores, min week^−1^	−0.772^**^	−0.772^**^	0.738^**^	0.734^**^	−0.738^**^	0.306^**^	0.312^**^	−0.430^**^	−0.689^**^	−0.746^**^	−0.585^**^	−0.705^**^

§ Data represent Rank-Biserial *r_rb_* correlation coefficients in case of dichotomous variables (otherwise - data represent Spearman's correlation coefficients - *r_s_*).

# Data represent Phi *φ* correlation coefficients in case of dichotomous variables (otherwise - data represent Rank-Biserial correlation coefficients - *r_rb_*).

* p<0.05; correlation is significant at the 0.05 level (2-tailed).

** p<0.01; correlation is significant at the 0.01 level (2-tailed).

(Underlined coefficients represent those which remained significant after the Bonferroni's corrections for multiple correlations, p<0.00003472). BCTQ – Boston Carpal Tunnel Questionnaire; CTS – carpal tunnel syndrome; EDX – electrodiagnostic testing; FSS – Functional Status Scale; MCV – motor conduction velocity; METs – metabolic equivalents; SCV – sensory conduction velocity; SDOL – sensory distal onset latency; SDOL-R – ratio of sensory distal onset latency; SSS – Symptom Severity Scale

The SSS, FSS, and EDX scores highly correlate with temporary/permanent work disability and certain clinical characteristics of CTS, including duration, bilateral presentation, family history, treatment, as well as with female gender, age, smoking, alcohol consumption, physical inactivity, BMI, systolic and diastolic blood pressure, and diseases of endocrine glands (e.g., diabetes, thyroid disease). EDX findings of the ulnar nerve on the ring finger (but not on the little finger and hypothenar) show the same correlations but to a lesser extent (Table 6). In addition, BMI highly correlates with blood pressure, physical inactivity, smoking, alcohol consumption, overall health status, and endocrine glands disorders (p<0.001 for all, data not shown).

Table 7 shows a highly significant correlation between hours/months of work with computer and the severity of clinical impairments and temporary or permanent work disability. In addition, time spent daily at computer outside the workplace also highly correlates with these indicators. In contrast, the number and total duration of additional breaks, use of an ergonomically designed devices during work on the computer, hand exercises during breaks at work and outside work, and having completed the training for safe and healthy work with computers all highly negatively correlate with BCTQ and EDX findings and work disability. Other occupational factors unrelated to computer work that highly correlate with the severity of impairments and work disability include other repetitive jobs with hands, exposure to vibrations and its duration, work at low temperature, high air humidity, and draft, and low job satisfaction. As for the ulnar nerve on the ring finger EDX findings, they to correlate with the above factors but to a lesser extent.

**Table 7:** Correlations of indicators of subjective and objective impairments (BCSQ and EDX results) and work disability related to CTS with occupational factors related or not related to work with computer in the cohort of workers who work with a computer (N=190)

**Variable**	**Boston Carpal Tunnel Questionnaire (BCTQ)**		**Electrodiagnostic (EDX) testing**							**Work disability related to CTS**		
	**SSS score**	**FSS score**	**Median nerve MCV**	**Median nerve SCV**	**Median nerve SDOL**	**Ulnar nerve MCV**	**Ulnar nerve SCV**	**Ulnar nerve SDOL**	**Median / ulnar nerve SDOL-R**	**Temporary work disability duration**	**Permanent work disability application**	**Recommended job change**
	*r_s_ / r_rb_* ^§^	*r_s_ / r_rb_* ^§^	*r_s_ / r_rb_* ^§^	*r_s_ / r_rb_* ^§^	*r_s_ / r_rb_* ^§^	*r_s_ / r_rb_* ^§^	*r_s_ / r_rb_* ^§^	*r_s_ / r_rb_* ^§^	*r_s_ / r_rb_* ^§^	*r_s_ / r_rb_* ^§^	*r_rb_ / φ^#^*	*r_rb_ / φ ^#^*
**Occupational factors – computer work related**												
Duration of occupational work with computer, months	0.842^**^	0.855^**^	−0.990^**^	−0.988^**^	0.985^**^	−0.201^**^	−0.183^**^	0.425^**^	0.898^**^	0.990^**^	0.689^**^	0.675^**^
Average daily time spent in occupational work with computer, minutes	0.844^**^	0.856^**^	−0.984^**^	−0.983^**^	0.984^**^	−0.193^**^	−0.175^**^	0.415^**^	0.880^**^	0.988^**^	0.698^**^	0.676^**^
Total time in occupational computer work (minutes × months)	0.841^**^	0.853^**^	−0.988^**^	−0.986^**^	0.985^**^	−0.196^**^	−0.178^*^	0.421^**^	0.982^**^	0.989^**^	0.689^**^	0.673^**^
Average daily time spent in non-occupational work with computer, minutes	0.855^**^	0.869^**^	−0.987^**^	−0.985^**^	0.983^**^	−0.203^**^	−0.184^*^	0.424^**^	0.893^**^	0.987^**^	0.703^**^	0.687^**^
Number of additional breaks while occupationally working with computer	−0.721^**^	−0.751^**^	0.823^**^	0.821^**^	−0.826^**^	0.233^**^	0.217^**^	−0.409^**^	−0.779^**^	−0.826^**^	−0.591^**^	−0.683^**^
Total duration of additional breaks while occupationally working with computer, minutes	−0.728^**^	−0.760^**^	0.829^**^	0.827^**^	−0.832^**^	0.238^**^	0.223^**^	−0.417^**^	−0.782^**^	−0.833^**^	−0.589^**^	−0.681^**^
Exercises (finger and thumb stretching and wrist and hand joints rotations) during breaks at work	−0.718^**^	−0.736^**^	0.614^**^	0.611^**^	−0.617^**^	0.228^**^	0.222^**^	−0.312^**^	−0.594^**^	−0.625^**^	−0.571^**^	−0.667^**^
Exercises (finger and thumb stretching and wrist and hand joints rotations) during breaks outside of work	−0.769^**^	−0.781^**^	0.663^**^	0.660^**^	−0.662^**^	0.238^**^	0.232^**^	−0.359^**^	−0.648^**^	−0.668^**^	−0.514^**^	−0.602^**^
Use of a mouse pad while occupationally working with computer	0.016	0.014	−0.223^**^	−0.225^**^	0.229^**^	0.172^**^	0.184^**^	0.052	0.186^**^	0.217^**^	0.099	0.032
Use of an ergonomically designed mouse, mouse pad, and keyboard (adapted to the anatomical characteristics of the user's hand) while occupationally working with computer	−0.773^**^	−0.811^**^	0.649^**^	0.649^**^	−0.649^**^	0.258^**^	0.252^**^	−0.369^**^	−0.644^**^	−0.660^**^	−0.531^**^	−0.621^**^
Completed training for safe and healthy work with computers	−0.742^**^	−0.770^**^	0.598^**^	0.598^**^	−0.596^**^	0.249^**^	0.243^**^	−0.347^**^	−0.574^**^	−0.609^**^	−0.529^**^	−0.645^**^
**Occupational factors – not computer work related**												
Other repetitive jobs with hands at the workplace not related to computer-work	0.772^**^	0.757^**^	−0.691^**^	−0.685^**^	0.685^**^	−0.234^**^	−0.221^**^	0.418^**^	0.646^**^	0.685^**^	0.600^**^	0.688^**^
Other repetitive jobs with hands outside the workplace	0.197^**^	0.211^**^	−0.137^*^	−0.130^*^	0.120^*^	−0.094	−0.092	0.313^**^	0.151^*^	0.147^*^	0.257^**^	0.373^**^
Heavy physical exertion with arms/hands at the workplace outside the computer-work	0.043	0.063	0.038	0.043	−0.025	−0.184^**^	−0.185^**^	0.091	−0.008	−0.024	−0.004	−0.002
Heavy physical exertion with arms/hands outside the workplace	0.040	0.042	0.083	0.089	−0.088	−0.266^**^	−0.271^**^	0.081	−0.060	−0.076	−0.052	0.028
Exposure to vibrations at the workplace	0.772^**^	0.780^**^	−0.741^**^	−0.736^**^	0.746^**^	−0.198^**^	−0.186^*^	0.442^**^	0.697^**^	0.750^**^	0.576^**^	0.670^**^
Duration of exposure to vibrations at the workplace, minutes	0.795^**^	0.802^**^	−0.772^**^	−0.768^**^	0.779^**^	−0.119	−0.105	0.443^**^	0.719^**^	0.781^**^	0.714^**^	0.766^**^
The level of vibration at the workplace above limit	0.042	0.089	−0.047	−0.043	0.042	−0.005	−0.010	−0.018	0.070	0.057	0.096	0.204^**^
Exposure to vibrations out of work	0.795^**^	0.802^**^	−0.772^**^	−0.768^**^	0.779^**^	−0.119	−0.105	0.443^**^	0.719^**^	0.781^**^	0.714^**^	0.766^**^
Air temperature at the workplace												
Normal	−0.605^**^	−0.613^**^	0.665^**^	0.664^**^	−0.664^**^	0.043	0.035	−0.105	−0.557^**^	−0.665^**^	−0.612^**^	−0.353^**^
Increased	0.056	0.073	−0.146^*^	−0.144^*^	0.147^*^	−0.078	−0.077	0.021	0.153^*^	0.145^*^	0.002	−0.102
Decreased	0.609^**^	0.612^**^	−0.640^**^	−0.640^**^	0.639^**^	−0.018	−0.010	0.102	0.526^**^	0.641^**^	0.634^**^	0.401^**^
Air temperature at the workplace, degrees Celsius	−0.850^**^	−0.856^**^	0.985^**^	0.983^**^	−0.982^**^	0.176^**^	0.158^*^	−0.418^**^	−0.891^**^	−0.988^**^	−0.701^**^	−0.681^**^
Relative air humidity at the workplace												
Normal	−0.772^**^	−0.767^**^	0.715^**^	0.710^**^	−0.714^**^	0.254^**^	0.241^**^	−0.418^**^	−0.667^**^	−0.720^**^	−0.555^**^	−0.666^**^
Decreased	0.056	0.073	−0.146^*^	−0.144^*^	0.147^*^	−0.078	−0.077	0.021	0.153^*^	0.145^*^	0.002	−0.102
Increased	0.758^**^	0.748^**^	−0.678^**^	−0.674^**^	0.677^**^	−0.234^**^	−0.222^**^	0.413^**^	0.628^**^	0.683^**^	0.554^**^	0.691^**^
Air-flow speed at the workplace												
Normal	−0.778^**^	−0.769^**^	0.735^**^	0.730^**^	−0.735^**^	0.222^**^	0.210^**^	−0.416^**^	−0.687^**^	−0.732^**^	−0.584^**^	−0.653^**^
Decreased	0.056	0.073	−0.146^*^	−0.144^*^	0.147^*^	−0.078	−0.077	0.021	0.153^*^	0.145^*^	0.002	−0.102
Increased	0.763^**^	0.751^**^	−0.698^**^	−0.694^**^	0.698^**^	−0.203^**^	−0.191^**^	0.410^**^	0.648^**^	0.696^**^	0.584^**^	0.678^**^
General job satisfaction	−0.738^**^	−0.750^**^	0.593^**^	0.590^**^	−0.596^**^	0.225^**^	0.229^**^	−0.327^**^	−0.573^**^	−0.602^**^	−0.534^**^	−0.648^**^
Poorly satisfied	0.740^**^	0.749^**^	−0.581^**^	−0.578^**^	0.584^**^	−0.220^**^	−0.215^**^	0.324^**^	0.564^**^	0.589^**^	0.542^**^	0.659^**^
Moderately satisfied	−0.610^**^	−0.608^**^	0.443^**^	0.441^**^	−0.447^**^	0.168^*^	0.142^*^	−0.257^**^	−0.435^**^	−0.446^**^	−0.461^**^	−0.567^**^
Very satisfied	−0.296^**^	−0.315^**^	0.298^**^	0.298^**^	−0.297^**^	0.114	0.152^*^	−0.147^*^	−0.280^**^	−0.309^**^	−0.189^**^	−0.219^**^

§ Data represent Rank-Biserial *r_rb_* correlation coefficients in case of dichotomous variables (otherwise - data represent Spearman's correlation coefficients - *r_s_*).

# Data represent Phi φ correlation coefficients in case of dichotomous variables (otherwise - data represent Rank-Biserial correlation coefficients - *r_rb_*).

* p<0.05; correlation is significant at the 0.05 level (2-tailed).

** p<0.01; correlation is significant at the 0.01 level (2-tailed).

(Underlined coefficients represent those which remained significant after the Bonferroni's corrections for multiple correlations, p<0.00003472). BCTQ – Boston Carpal Tunnel Questionnaire; CTS – carpal tunnel syndrome; EDX – electrodiagnostic testing; FSS – Functional Status Scale; MCV – motor conduction velocity; SCV – sensory conduction velocity; SDOL – sensory distal onset latency; SDOL-R – ratio of sensory distal onset latency; SSS – Symptom Severity Scale

Since many participants were exposed to combined occupational risks significantly correlating with CTS (vibrations, other repetitive jobs with hands not related to work with computer, work under decreased air temperature, increased air relative humidity, and increased air-flow speed), we repeated the correlation analysis in a subgroup of workers unexposed to additional occupational risks i.e., in those who worked under normal microclimatic conditions, without vibrations, heavy physical exertion, or other repetitive jobs with hands (N=83) only to find even more significant correlations between some occupational factors related to computer work and the severity of objective clinical manifestations (EDX results) or temporary work disability indicators but a bit less significant correlation with the severity of subjective clinical manifestations (BCTQ results), since their BCTQ scores are generally lower (the median score is 1 on both BCTQ scales) (data not shown). More specifically, EDX results on the median nerve and temporary work disability (with *rs* correlations coefficients ranging from 0.839 to 0.975; p<0.001) significantly correlate with the duration of work on a computer and use of additional breaks, which has remained significant even after the Bonferroni correction. However, we have found no correlation with permanent work disability indicators.

Likewise, the subgroup of the remaining workers (N=107), those with additional occupational risk-factors, shows a significant correlation between the duration of computer work with objective CTS manifestations and work disability (*r_s_* ranging from 0.950 to 0.973; p<0.001) or subjective (BCTQ) scores (*r_s_* ranging from 0.840 to 0.880; p<0.001) after the Bonferroni correction.

Furthermore, workers without additional occupational risk factors (N=83) scored much lower on BCTQ and EDX than those with additional risks (N=107) and have a lower rate of temporary and permanent work disability (Mann-Whitney *U* test and chi-squared test, p<0.001 for all, data not shown), which points to an additive (or even synergistic) effect of occupational risk factors, whether related to computer work or not.

Table 8 clearly shows that foods clustered in “dietary cluster 1” positively correlate with the level of impairments and work disability, whereas foods clustered in “dietary cluster 2” and supplementation correlate negatively. The same goes for the correlation of dietary clusters and supplementation with the BMI: “dietary cluster 1” shows a strong positive while “dietary cluster 2” and supplementation a strong negative correlation (p<0.001 for all, data not shown).

**Table 8:** Correlations of indicators of subjective and objective impairments (BCSQ and EDX results) and work disability related to CTS with frequency of consumption of certain food groups and dietary supplements in the cohort of workers who work with a computer (N=190)

**Variable**	**Boston Carpal Tunnel Questionnaire (BCTQ)**		**Electrodiagnostic (EDX) testing**							**Work disability related to CTS**		
	**SSS score**	**FSS score**	**Median nerve MCV**	**Median nerve SCV**	**Median nerve SDOL**	**Ulnar nerve MCV**	**Ulnar nerve SCV**	**Ulnar nerve SDOL**	**Median / ulnar nerve SDOL-R**	**Temporary work disability duration**	**Permanent work disability application**	**Recommended job change**
	*r_s_* ^§^	*r_s_* ^§^	*r_s_* ^§^	*r_s_* ^§^	*r_s_* ^§^	*r_s_* ^§^	*r_s_* ^§^	*r_s_* ^§^	*r_s_* ^§^	*r_s_* ^§^	*r_rb_ ^#^*	*r_rb_^#^*
**Frequency of food groups and dietary supplements consumption**												
Frequency of consumption of refined grain products, red and processed meat, tallow, sweets, dried fruits, carbonated sugar-added beverages and spirits (dietary cluster 1)	0.621^**^	0.620^**^	0.531^**^	0.539^**^	−0.551^**^	−0.289^**^	−0.283^**^	0.194^**^	0.554^**^	0.564^**^	0.453^**^	0.412^**^
Frequency of consumption of integral grain products, eggs, poultry, fish and sea products, fresh vegetables, fresh fruits and fruit-juices, nuts, vegetable oils (including sunflower oil and olive oil), pork lard, and beer (dietary cluster 2)	−0.748^**^	−0.721^**^	0.723^**^	0.722^**^	−0.723^**^	0.298^**^	0.287^**^	−0.343^**^	−0.725^**^	−0.731^**^	−0.550^**^	−0.607^**^
Frequency of use of all dietary supplements (all examined supplements included)	−0.800^**^	−0.806^**^	0.847^**^	0.844^**^	−0.848^**^	0.285^**^	0.274^**^	−0.410^**^	−0.843^**^	−0.852^**^	−0.595^**^	−0.677^**^

§ Data represent Spearman's correlation coefficients - *r_s_*

# Data represent Rank-Biserial correlation coefficients - *r_rb_*

* p<0.05; correlation is significant at the 0.05 level (2-tailed).

** p<0.01; correlation is significant at the 0.01 level (2-tailed).

(Underlined coefficients represent those which remained significant after the Bonferroni's corrections for multiple correlations, p<0.00003472). BCTQ – Boston Carpal Tunnel Questionnaire; CTS – carpal tunnel syndrome; EDX – electrodiagnostic testing; FSS – Functional Status Scale; MCV – motor conduction velocity; SCV – sensory conduction velocity; SDOL – sensory distal onset latency; SDOL-R – ratio of sensory distal onset latency; SSS – Symptom Severity Scale

As for laboratory findings (Table 9), all inflammation and metabolic impairment markers but AST highly correlate with measures of CTS severity and work disability. As expected, they also highly correlate with BMI, save for AST (p *<*0.001 for all, data not shown).

**Table 9:** Correlations of indicators of subjective and objective impairments (BCSQ and EDX results) and work disability related to CTS with laboratory markers of inflammation and metabolic risk in the cohort of workers who work with a computer (N=190)

**Variable**	**Boston Carpal Tunnel Questionnaire (BCTQ)**		**Electrodiagnostic (EDX) testing**							**Work disability related to CTS**		
	**SSS score**	**FSS score**	**Median nerve MCV**	**Median nerve SCV**	**Median nerve SDOL**	**Ulnar nerve MCV**	**Ulnar nerve SCV**	**Ulnar nerve SDOL**	**Median / ulnar nerve SDOL-R**	**Temporary work disability duration**	**Permanent work disability application**	**Recommended job change**
	*r_s_* ^§^	*r_s_* ^§^	*r_s_* ^§^	*r_s_* ^§^	*r_s_* ^§^	*r_s_* ^§^	*r_s_* ^§^	*r_s_* ^§^	*r_s_* ^§^	*r_s_* ^§^	*r_rb_ ^#^*	*r_rb_^#^*
**Laboratory biochemical indicators of metabolic risk and inflammation**												
LDL-cholesterol	0.829^**^	0.851^**^	−0.980^**^	−0.980^**^	0.972^**^	−0.190^**^	−0.173^*^	0.424^**^	0.891^**^	0.976^**^	0.689^**^	0.672^**^
HDL-cholesterol	−0.847^**^	−0.856^**^	0.944^**^	0.942^**^	−0.932^**^	0.175^*^	0.162^*^	−0.415^**^	−0.848^**^	−0.938^**^	−0.668^**^	−0.653^**^
Triglycerides	0.850^**^	0.864^**^	−0.839^**^	−0.835^**^	0.830^**^	−0.182^*^	−0.173^*^	0.403^**^	0.738^**^	0.836^**^	0.670^**^	0.687^**^
Glucose	0.767^**^	0.772^**^	−0.678^**^	−0.674^**^	0.686^**^	−0.124	−0.114	0.318^**^	0.613^**^	0.705^**^	0.561^**^	0.600^**^
AST	0.041	−0.015	0.059	0.061	−0.047	−0.021	−0.024	0.062	−0.015	−0.055	−0.160^*^	−0.115
ALT	0.414^**^	0.395^**^	−0.358^**^	−0.354^**^	0.366^**^	−0.079	−0.066	0.175^*^	0.284^**^	0.349^**^	0.381^**^	0.333^**^
GGT	0.275^**^	0.308^**^	−0.195^**^	−0.188^**^	0.188^**^	0.008	0.027	0.017	0.183^*^	0.201^**^	0.258^**^	0.244^**^
Uric acid	0.593^**^	0.583^**^	−0.472^**^	−0.470^**^	0.470^**^	−0.152^*^	−0.144^*^	0.268^**^	0.512^**^	0.465^**^	0.445^**^	0.543^**^
Urea	0.702^**^	0.705^**^	−0.668^**^	−0.666^**^	0.653^**^	−0.064	−0.047	0.443^**^	0.635^**^	0.660^**^	0.531^**^	0.685^**^
Creatinine	0.601^**^	0.622^**^	−0.639^**^	−0.640^**^	0.624^**^	−0.237^**^	−0.221^**^	0.353^**^	0.576^**^	0.640^**^	0.446^**^	0.604^**^
ESR	0.846^**^	0.862^**^	−0.833^**^	−0.832^**^	0.824^**^	−0.151^*^	−0.140	0.376^**^	0.803^**^	0.837^**^	0.710^**^	0.757^**^
Fibrinogen	0.683^**^	0.712^**^	−0.611^**^	−0.613^**^	0.612^**^	−0.176^*^	−0.164^*^	0.291^**^	0.603^**^	0.617^**^	0.585^**^	0.627^**^
CRP	0.852^**^	0.869^**^	−0.973^**^	−0.973^**^	0.975^**^	−0.199^**^	−0.183^*^	0.405^**^	0.891^**^	0.977^**^	0.701^**^	0.666^**^

§ Data represent Spearman's correlation coefficients - *r_s_*

# Data represent Rank-Biserial correlation coefficients - *r_rb_*

* p<0.05; correlation is significant at the 0.05 level (2-tailed).

** p<0.01; correlation is significant at the 0.01 level (2-tailed).

(Underlined coefficients represent those which remained significant after the Bonferroni's corrections for multiple correlations, p<0.00003472). ALT – alanine aminotransferase; AST – aspartate aminotransferase; BCTQ – Boston Carpal Tunnel Questionnaire; CRP – C-reactive protein; CTS – carpal tunnel syndrome; EDX – electrodiagnostic testing; ESR – erythrocyte sedimentation rate; FSS – Functional Status Scale; GGT – gamma-glutamyl transferase; HDL – high-density lipoprotein; LDL – low-density lipoprotein; MCV – motor conduction velocity; SCV – sensory conduction velocity; SDOL – sensory distal onset latency; SDOL-R – ratio of sensory distal onset latency; SSS – Symptom Severity Scale

## DISCUSSION

Our study clearly identifies occupational factors of computer work that aggravate CTS-related subjective and objective symptoms and contribute to permanent and temporary work disability, including the total duration of computer work, inappropriate ergonomic conditions, insufficient number and duration of breaks, failing to do hand exercises, and lack of adequate training for computer work, confirming a number of previous reports (3, 4, 5, 7, 10, 61, 62, 63). Considering that these factors can be changed, our findings call on both employers and employees to take preventive/corrective measures and improve working conditions and safety at work.

In addition, we have identified occupational factors unrelated to computer work (including other repetitive jobs with hands, vibrations, improper microclimatic conditions) that can aggravate CTS-related symptoms and are in line with previous reports (1, 25, 29, 32, 40, 64, 65, 66, 67). They seem to have additive (or even synergistic) effects to those related to computer work and call for further studies. However, we did not find any correlation with heavy physical exertion with arms/hands, a factor often mentioned in the literature (28, 29, 32, 33, 40, 67), most probably because not many study participants did heavy work with arms/hands.

As expected from earlier reports (39, 66), psychological factors such as low job satisfaction correlate significantly with aggravated CTS symptoms and work disability. Education, in turn, does not, although we see a trend towards a negative correlation, which is in agreement with previously reported data (5).

Regarding non-occupational risk factors, our study has identified a number of them as contributing to the severity of CTS symptoms that have been reported earlier, including female gender, age, family history, BMI, smoking, alcohol consumption, physical inactivity (35, 36, 38, 39, 63, 68, 69, 70, 71, 72), diabetes and hypothyroidism (36, 38, 73, 74, 75, 76, 77), hypertension (38, 71, 78), and consistently, laboratory markers of metabolic disorders and inflammation, although these have rarely been studied in this context (78). Still, the mechanisms by which obesity, diabetes, hypothyroidism, hypertension, and smoking may aggravate CTS are still unclear (38, 68, 71, 75, 78, 79, 80, 81, 82, 83, 84, 85, 86, 87, 88, 89, 90, 91).

Rare are also the studies directly associating dietary habits with CTS, such as the one published by Lin (92), but there are many associating them with musculoskeletal disorders, inflammation, and obesity (93, 94, 95, 96, 97, 98, 99, 100). Our study therefore brings new evidence of the correlation between unhealthy, energy-dense, nutrient-poor diet and aggravated clinical symptoms of CTS and work disability. It is well known that physical inactivity, unhealthy diet, hypertension, and laboratory markers of inflammation and metabolic disorders are associated with increased BMI (101, 102, 103), which and may indirectly aggravate CTS through increased BMI (36, 72, 79, 104), although direct effects cannot be excluded (77, 93, 94, 95, 97, 105). The same goes for supplements; it is unclear if the correlations found are confounded by healthy lifestyle and normal BMI or there are more direct effects of certain micronutrients, as suggested by some studies (37, 93, 97, 105, 106, 107, 108, 109, 110, 111, 112, 113, 114, 115, 116, 117, 118, 119). Therefore, further research is needed to establish a direct cause-effect relationship.

Another novelty of our study is the analysis of CTS-related work disability among workers who use computers at work. CTS is a common cause of sick leave, disability pension, and compensation claims, creating a substantial economic burden (26, 40, 120). However, we found no compensation claims raised on account of CTS in North Macedonia. Instead, our cohort resorted to taking sick leave (about one month in average over the past year), about 40 % were advised to change the job, and one-third applied for permanent work disability pension, although only a few were granted one (post-study information). As we could not find disability studies covering a similar working population on computers, we can only compare our data with the other working populations of other countries (120, 121, 122, 123, 124, 125). In general, data on working disability related to CTS are scarce, and mainly concern those collected after surgical interventions (126, 127, 128, 129). Moreover, these data are hardly comparable as health insurance policies and compensations in other countries may differ from those in North Macedonia (122, 127, 129). The median work time lost to CTS in our study is 31 days a year, which is a bit higher than in the United States (25 days) (130), but it could reflect different health insurance policies, not only less efficient medical treatment or prevention (131). As expected, participants with longer sick leave applied for permanent work disability or were advised to change the job more often than the rest, which is in line with other reports (122). Furthermore, longer sick leave correlates with worse subjective and objective manifestations, bilateral presentation, physical therapy, use of steroidal and non-steroidal anti-inflammatory drugs, and, above all, surgery, female gender, family history, endocrine diseases, increased BMI, physical inactivity smoking, alcohol use, unhealthy diet, and no supplement use, which confirms scant reports for other working populations (26, 41, 120, 122) or patients with related surgical interventions (127, 128, 129).

We also found a correlation between the sensory recordings on the ulnar nerve on the ring finger and CTS aggravation, disability, and risk factors, which supports other studies evidencing common moderate involvement of the ulnar nerve in the clinical presentations of CTS (132, 133, 134, 135, 136, 137, 138) or that computer work is associated with the ulnar nerve entrapment at the elbow (cubital tunnel syndrome) and the wrist (Guyon's canal syndrome) due to repetitive compression from leaning on the elbows or wrists during work with a mouse or a keyboard (139, 140, 141).

What is a unique strength of this study is the relatively large cohort and an encompassing analysis of many different risk factors for CTS development and aggravation. However, there are some limitations too, which need addressing. Even though our sample size meets statistical requirements (53), it may not ensure sufficient statistical power for the multiple statistical tests performed here, subgroup analyses in particular (54). We, however, believe to have overcome this limitation by resorting to the Bonferroni correction to avoid bias pertinent to multiple correlation testing that may result in type I error (58). Furthermore, although we have found significant correlations, the cross-sectional design of this study does not allow inferring any direct cause-and-effect relationships. It is also a highly possible that the established correlations have been confounded by other factors (e.g., the relationship between BMI and physical inactivity, improper diet, blood pressure, and laboratory markers or the relationships between computer work and vibrations, other repetitive movements, and negative microclimatic conditions or the relationships between duration of work with computer and age and duration of illness). In other words, we cannot claim that these factors had an independent effect. The effects could have been additive or even synergistic, and we cannot distinguish the influence of the confounding correlations. To establish independent effects of specific factors, further research should resort to partial correlations and multiple linear regression analyses, which calls for a much larger sample to accommodate a great number of possible covariates. To study possible causality, a case-control study with multiple logistic regression would be a better design choice.

Some may also question the validity of the Macedonian BCTQ version, as it had not been validated in the Macedonian population before we used it, but we have established a high correlation between BCTQ scores and EDX results for the median nerve, with Spearman's *r_s_* correlation coefficients ≥0.840, which is even higher than in some previous validation studies (42, 59, 142). This indicates that BCTQ, as a measure of subjective symptoms, correlates well with objective impairments, and confirms its construct validity (142).

Finally, our study is not limited to workers who exclusively work on the computer all the working time over several years, but this has allowed us to see how the duration of computer work correlates with objective and subjective CTS symptoms. Besides, the median time spent on computer work in our study is 4 h per day, which is well above or around the time associated with the increased risk of CTS reported by some studies (ranging from 2 h and 15 min per day to 4 h per day or 20 h per week) (2, 3, 4, 7, 8, 62). In fact, 77.9 % of our participant reported working with computer at least 2 h and 15 min per day.

The public health significance of CTS is high, having in mind the prevalence among active workers (5, 41, 143) and the socioeconomic burden (41, 120, 122, 125, 144, 145). This study identifies areas that need improvement in real-life situations at work, starting from better ergonomic measures and training in this sense to raising awareness about the importance of adequate duration of work with computer, regular breaks, hand exercises, favourable microclimatic conditions, and minimal exposure to vibrations (146, 147). Furthermore, our study points to a number of non-occupational risks that can be addressed, most notably unhealthy diet and lifestyle, through education and regular check-ups.

In conclusion, even though our results need to be interpreted with caution due to methodological limitations, our study clearly identifies occupational and non-occupational risk factors that can lead to or aggravate CTS. More research is needed, though, to establish possible causal relationships and the independent influence of each of these risk factors.
